# Telomerase inhibition improves tumor response to radiotherapy in a murine orthotopic model of human glioblastoma

**DOI:** 10.1186/s12943-015-0376-3

**Published:** 2015-07-17

**Authors:** Sylvain Ferrandon, Céline Malleval, Badia El Hamdani, Priscillia Battiston-Montagne, Radu Bolbos, Jean-Baptiste Langlois, Patrick Manas, Sergei M Gryaznov, Gersende Alphonse, Jérôme Honnorat, Claire Rodriguez-Lafrasse, Delphine Poncet

**Affiliations:** EMR3738, Cellular and Molecular Radiobiology Laboratory, South Lyon Charles Mérieux Medicine Faculty, Oullins, France; Université Claude Bernard Lyon 1, Lyon, France; Team « neuro-oncology and neuro-inflammation », Lyon Neuroscience Research Center, INSERM U1028/CNRS UMR 5292, Laennec Medicine Faculty, Lyon, France; Hospices Civils de Lyon, South Lyon Hospital, Pierre Bénite, France; CERMEP-imagerie du vivant, Groupement Hospitalier Est, Bron, France; UMS 3444 gerland CNRS P.B.E.S - Ecole Normale Supérieure Lyon, Lyon, France; Geron Corporation, 149 Commonweath Drive, Menlo, Park, CA 94025 USA; Department of neuro-oncology, Hospices civils de Lyon, Bron, France; Biochemistry Department, Transfer and Molecular Oncology Unit, South Lyon Hospital, Hospices Civils de Lyon, Pierre Bénite, France

**Keywords:** Telomerase, Glioma, Irradiation, Orthotopic, GRN163L, Imetelstat, In vivo

## Abstract

**Background:**

Glioblastoma (GBM) is the most frequent and aggressive type of adult brain tumor. Most GBMs express telomerase; a high level of intra-tumoral telomerase activity (TA) is predictive of poor prognosis. Thus, telomerase inhibitors are promising options to treat GBM. These inhibitors increase the response to radiotherapy (RT), *in vitro* as well as *in vivo*. Since typical treatments for GBM include RT, our objective was to evaluate the efficiency of Imetelstat (TA inhibitor) combined with RT.

**Findings:**

We used a murine orthotopic model of human GBM (N = 8 to11 mice per group) and μMRI imaging to evaluate the efficacy of Imetelstat (delivered by intra-peritoneal injection) alone and combined with RT. Using a clinically established protocol, we demonstrated that Imetelstat significantly: (i) inhibited the TA in the very center of the tumor, (ii) reduced tumor volume as a proportion of TA inhibition, and (iii) increased the response to RT, in terms of tumor volume regression and survival increase.

**Conclusions:**

Imetelstat is currently evaluated in refractory brain tumors in young patients (without RT). Our results support its clinical evaluation combined with RT to treat GBM.

**Electronic supplementary material:**

The online version of this article (doi:10.1186/s12943-015-0376-3) contains supplementary material, which is available to authorized users.

## Findings

Reactivation of a telomeric maintenance mechanism is a key and obligatory step for all cancer cells. In fact, telomerase is reactivated in 96 % of cancer tumors. Thus, inhibition of this enzyme is a promising option for treating cancer [1]. GRN163L (Imetelstat) is the most advanced telomerase inhibitor in clinical evaluation (17 completed or ongoing clinical trials) (https://clinicaltrials.gov). In preclinical assays using numerous models of solid and liquid tumors, a reduction in tumor volume and an increase in overall survival (OS) have been reported [2].

In addition, most cancers are treated by radiotherapy (RT), and among the whole genome, γ-irradiation (IR) preferentially targets telomeres [3] and induces a reduction in telomere length [4]. Moreover, shortening telomeres [5–9] increases cell sensitivity to IR, whereas, elongating telomeres induces radio-resistance [5, 10]. Thus, it makes sense to combine Imetelstat with radiotherapy, as demonstrated in two murine models of breast cancer [6, 11].

Glioblastoma (grade IV astrocytoma)(GBM) is the most frequent adult brain tumor, and one of the most aggressive tumors among all human cancers. Despite a standard of care combining surgery, radiotherapy (RT) and Temozolomide chemotherapy, the median patient overall survival (OS) still ranges from 7 to 15 months [12]. New targeted therapies or irradiation techniques are thus urgently needed. However, identification of cancer-specific targets is a particular challenge in GBM as these tumors are highly heterogeneous in terms of their histological, molecular, genetic and epigenetic features [12]. However, most GBMs reactivate telomerase [13–15]. Among these tumors a high intra-tumoral level of telomerase activity (TA) [14, 15] is predictive of poor prognosis. Two mutations residing in the promoter of TERT (encoding the catalytic subunit of the telomerase) have recently been identified. These mutations, increasing the TA, have been detected in many types of tumors [16], with the highest frequency in GBM (74 % of patients). Telomerase inhibitors are thus a promising option for treating GBMs.

Drug delivery into the brain is challenging as the Blood Brain Barrier (BBB) stops most of the molecules. Hence, the two first preclinical studies evaluating Imetelstat, in an orthotopic location, delivered the drug directly into the brain [17, 18]. However, in high grade glioma the BBB is leaky [19], and a more recent publication has provided evidence indicating that Imetelstat passes the BBB [7]. It remains to be demonstrated that the reduction of TA is correlated with a clinical response (such as tumor growth reduction or OS increase). More importantly, RT is used in all GBM treatment plans and Marian et al. have shown, *in vitro*, on human GBM tumor-initiating cells that 16 weeks of pre-treatment by IMT increases cell death and DNA damage produced by IR. However, the *in vivo* efficacy of Imetelstat in combination with RT, using a realistic treatment plan, has never been evaluated in GBM.

In this article, we propose, using the intra-peritoneal route in a murine orthotopic model of human GBM, to: (i) demonstrate a reduction in tumor growth in relation with the level of TA inhibition inside the tumor and to (ii) evaluate if Imetelstat increases the efficiency of RT.

Considering the *in vivo* experiment, we chose a glioblastoma-derived cell line with detectable TA and a telomere length of about 4 kb (in human TA+ GBM, telomere length ranges from 2 to 11 kb [13]). This cell line, U87MG, is also the most suitable model for *in vivo* preclinical assays [20], as it gives rise to tumors in a very reproducible manner. All materials and methods are described in Additional file 1.

**Fig. 1:**
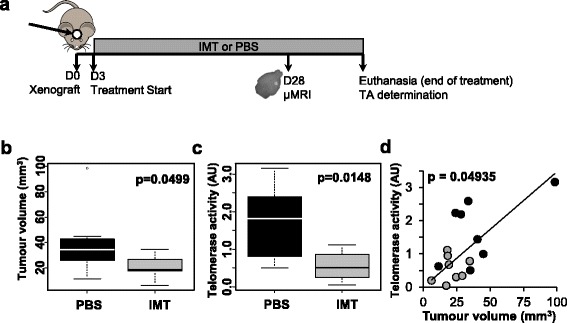
Intra-peritoneal injection of Imetelstat efficiently inhibits telomerase and reduces tumor growth. **a** Experimental design: mice were xenografted and intra-peritoneal injections were started three days later, either with Imetelstat (30 mg/Kg three times a week) or by an equivalent volume of PBS. Tumor volume was determined by μMRI at day 28, and the treatment was maintained until the mice were sacrificed (when tumor growth was predicted to be about 70 mm^3^ by the μMRI imaging). **b** Tumor volume at day 28 is significantly reduced by Imetelstat (IMT) treatment versus PBS (Wilcoxon test). **c** Intra-peritoneal injection of Imetelstat is able to significantly reduce the TA inside the tumor (Wilcoxon test). **d** TA and tumor volume are correlated (Spearman test), the grey and black circles correspond respectively to the mice treated by IMT or by PBS

First, we wanted to validate the efficiency of the drug alone, before combining it with RT. The U87MG cell line was xenografted in an orthotopic location, and mice were treated by Imetelstat (N = 8) or by the vehicle PBS (Phosphate Buffer Saline) (N = 8), by the intra-peritoneal route, from day 3 (post-xenograft) to euthanasia (Fig. 1a). Twenty-eight days post-graft, we noted a significant reduction in tumor volume (Fig. 1b) with the mice receiving Imetelstat, attesting, for the first time, to the treatment’s efficiency when using a peripheral route of injection. However, this efficiency was to be put in relation to the inhibition of the TA. Thus, we measured the TA in the very center of the tumor and observed a significant reduction (Fig. 1c). This confirms that Imetelstat efficiently reaches the center of the tumor. A significant and positive correlation between tumor growth and the residual level of TA was also shown (Fig. 1d). This observation proves: (i) that the anti-tumoral activity of Imetelstat is due to its anti-telomerase activity, and (ii) that TA plays an essential role in GBM growth and aggressiveness, reinforcing the interest in targeting telomerase to treat GBM.

**Fig. 2:**
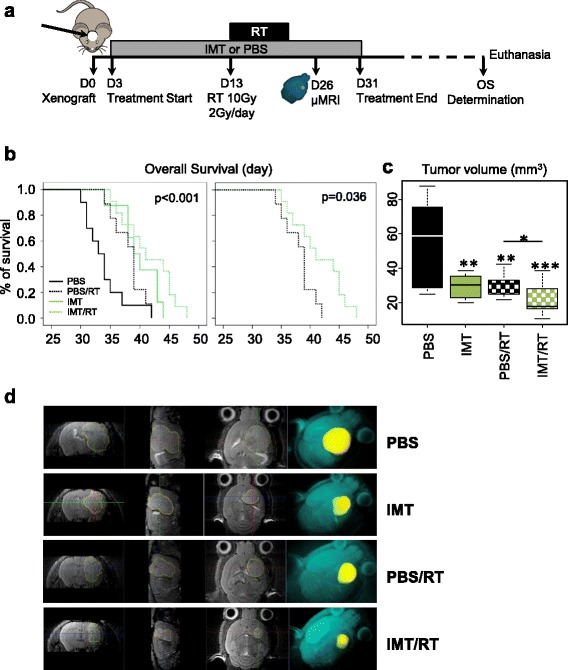
Imetelstat significantly increases radiotherapy efficiency *in vivo*. **a** Experimental design: mice were xenografted (D0) and intra-peritoneal injections were started three days later (D3) either with Imetelstat (IMT, 30 mg/Kg three times a week) or by an equivalent volume of PBS, for four weeks. Two weeks after the treatment was began (D13) mice were or were not concomitantly treated by radiotherapy (RT, 2Gy/day, five days), and imaged by μMRI at day 26 (D26). Injections were stopped at the end of the fourth week and mice were monitored until they developed debilitating disease (date used for the OS). **b** Kaplan Meier representation of OS as a function of days post-xenograft, for mice treated by PBS (black solid line), Imetelstat (green solid line) or a combination of RT with PBS (dashed black line) or with Imetelstat (dashed green line). LogRank calculated p-values are shown for all treatments (on the left) and for IMT/RT versus PBS/RT (on the right). **c** Tumor volume determined by μMRI at day 26 is shown for each treatment group. p-values were determined comparing each condition to the PBS condition, and between the PBS/RT versus the IMT/RT (p < 0.05, **p < 0.01, ***p < 0.001, Wilcoxon test). **d** Display of semi-automatic GBM segmentation on T2-weighted MR images: axial plane (left), sagittal plane (middle), coronal plane (right). A 3D reconstruction (right) showing the localization and the size of GBM (yellow) within the mouse brain (turquoise)

We next evaluated the efficiency of the combined treatment with RT, following a plan that would be suitable for human treatment. The mice were treated for one month with Imetelstat and RT was delivered concomitantly, two weeks post induction (as validated by our *in vitro* results, data not shown). The RT protocol was a focalized IR of the brain, five times a week by 2Gy fractions (as used for humans) for one week (Fig. 2a). On day 26, a significant reduction in tumor volume was observed by μMRI, in comparison with the PBS control group (PBS), regardless of the treatment : Imetelstat (IMT, p = 0.0084), PBS plus RT (PBS/RT, p = 0.0053), or Imetelstat plus RT (IMT/RT, p = 0.0004) (Figs. 2c, d). As observed in our *in vitro* experiments (data not shown), we noted that Imetelstat significantly increased the efficiency of RT, in term of tumor volume reduction (p = 0.0414) (Figs. 2c, d). As expected, the OS was increased in all 3 treatments (PBS/RT, IMT or IMT/RT) (Fig. 2b left). If considering the IMT/RT versus the PBS/RT groups we also established a significant (p = 0.036) increase in OS (Fig. 2b right). The median OS was respectively 30, 39, 39 and 41 days for the PBS, IMT, RT and IMT/RT conditions. Thanks to a standard curve of tumoral growth (Additional file 2: Figure S1), we have translated these results into tumoral volume variation: the combined treatment reduced the growth by 34 % in comparison to IMT or RT alone. Furthermore, 45 % of mice (5 over 11 mice) in the IMT/RT group have the same or an increased OS than mice in the RT or IMT group. To conclude, the combination of RT with Imetelstat significantly reduced tumor volumes and increased the OS of the mice.

Considering the results obtained with Imetelstat alone, we showed a significant reduction in TA, in the very center of the tumor, and established that the residual TA was positively correlated with the tumor’s growth. This highlights the major role of TA in these tumors’ aggressiveness in agreement with previous publications setting a correlation between patient outcome and tumoral-TA level [14, 15]. In the future, dose optimization could be proposed to patients, in proportion to the initial TA level inside their tumors or their circulating tumoral cells [21]. While, RT is an obligatory step for GBM treatment, no pre-clinical data was available concerning the combination of RT and Imetelstat for GBM treatment. We have demonstrated a significant improvement of the efficiency of RT, by combining it with Imetelstat for GBM treatment, in terms of OS increase and tumor volume reduction. It is noteworthy that we have used RT for only one week to avoid the radical effect of intensive RT that would have precluded any evaluation of its combined effect with Imetelstat (as in Serrano et al. publication [8]). Thus, we expect that the effect of the combination will be even more drastic with several weeks of concomitant RT (as commonly delivered in human GBM treatment). Moreover, we did not observe any side effect, confirming the results of previous preclinical assays [2].

Last, a major point of our model is that it avoids most of the inconsistencies of previous publications, such as the long-term cell pre-treatment before engraftment [11, 22], or the concomitant treatment together with engraftment [18], both of which preclude tumor engraftment and development [23]. This anti-engraftment or anti-adhesive [24] effect could be related to the off-target effect of Imetelstat on cytoskeleton destructuration [25]. Moreover, we propose a realistic protocol for clinical use: (i) a peripheral route of injection and not a direct intra-cerebral [18], intra-nasal [17] or intra-tumoral [11] delivery, (ii) a classical one-month cycle of chemotherapy and (iii) a combined fractionated RT treatment (2Gy/fraction delivered locally). Overall, our data supports the usefulness of future clinical studies using Imetelstat combined with RT for the treatment of GBM. The recent opening of a clinical trial evaluating Imetelstat (alone) in refractory brain tumors in young patients comforts this conclusion (NCT01836549).

## Additional files

Additional file 1:
**Materials and methods.**
Additional file 2:
**Determination of tumor growth after U87 orthotopic xenoraft.**

## Abbreviations

GBM: Glioblastoma
TA: Telomerase activity
RT: Radiotherapy
OS: Overall survival
IR: γ-irradiation
IMT: Imetelstat
