# Supplementary material for: Telomerase inhibition improves tumor response to radiotherapy in a murine orthotopic model of human glioblastoma
Source: Mol Cancer. 2015 Jul 17;14:134. doi: 10.1186/s12943-015-0376-3 (PMC4504179; doi:10.1186/s12943-015-0376-3)
Supplement: Additional file 1: — Materials and methods. [file 12943_2015_376_MOESM1_ESM.docx]

**Additional file 1**: Materials and methods.

Our protocol has been approved by the local ethics committee, in accordance to the European guidelines at the Animal Laboratory Center of South Lyon (UMS3444/US8). Murine Orthotopic human GBM models were obtained by intra-cerebral injection of U87MG cells (purchased from ATCC) in Female athymic Nude mice (Charles Rivers, Arbresle, France), as previously described [1]. Treatments were driven as described in the experimental designs (Fig. 1a, 2a). Mice were sacrificed by decerebration. In the first experiment, the tumors were collected and immediately frozen in cold (-50°) isopentane and conserved at -80°C. Serial sections (30 µm thick) were cut with a cryostat until the center of the tumor was reached, and then a biopsy was collected from the center of the tumor, for the TRAP assay. A section stained with Hematoxylin & Eosin was done to confirm the biopsy location and the absence of necrosis. Brain-focalized irradiation (2Gy/day, five days per week for one week), were led under anesthesia (isoflurane) using an XRAD 320 at the UMS3444/US8 facilities. TA was measured using the TeloTAGGG Telomerase PCR ELISA kit, following the manufacturer’s instructions (Roche Applied Bioscience, Meylan, France). Tumors were dissected and lysed in the TRAP lysis buffer. Lysates were all diluted at the same concentration (0.002µg/µL) and 1µL of lysate was used in a TRAP reaction. Each sample was analyzed with or without heat inactivation (85°C for 10min). All the results were in the linear section of the dilution series.

*In vivo* MRI acquisitions were performed on a 7T Bruker MRI system (Bruker Biospin, Germany). A number of 30 coronal MR images of 220 µm slice thickness were acquired on mice brains using a T2-weighted RARE sequence, with 78 x 78 µm^2^ resolution, TR/TE = 5000/33.5 ms. The animals were anesthetized using 1.5 to 2.5% isoflurane gas, and their respiration rate was monitored during the entire scan. The MR images were then analyzed and the 3D volume was reconstructed (in mm^3^) by dedicated software (AMIRA, Visual Science Group, Burlington, MA, USA).

Considering the statistical analyses, we run Wilcoxon (mean comparison), Anova (comparison of two effects as a function of time), and Spearman correlation test with R software (R Development Core Team (2005), R Foundation for Statistical Computing, Vienna, Austria). The Kaplan Meier curves and LogRank tests were run on SPSS software.

1. Jarry M, Lecointre C, Malleval C, Desrues L, Schouft MT, Lejoncour V, Liger F, Lyvinec G, Joseph B, Loaec N, et al: **Impact of meriolins, a new class of cyclin-dependent kinase inhibitors, on malignant glioma proliferation and neo-angiogenesis.** *Neuro Oncol* 2014.
