# Supplementary material for: Telomerase inhibition improves tumor response to radiotherapy in a murine orthotopic model of human glioblastoma
Source: Mol Cancer. 2015 Jul 17;14:134. doi: 10.1186/s12943-015-0376-3 (PMC4504179; doi:10.1186/s12943-015-0376-3)
Supplement: Additional file 2: — Determination of tumor growth after U87 orthotopic xenoraft. [file 12943_2015_376_MOESM2_ESM.ppt]

## Slide 1
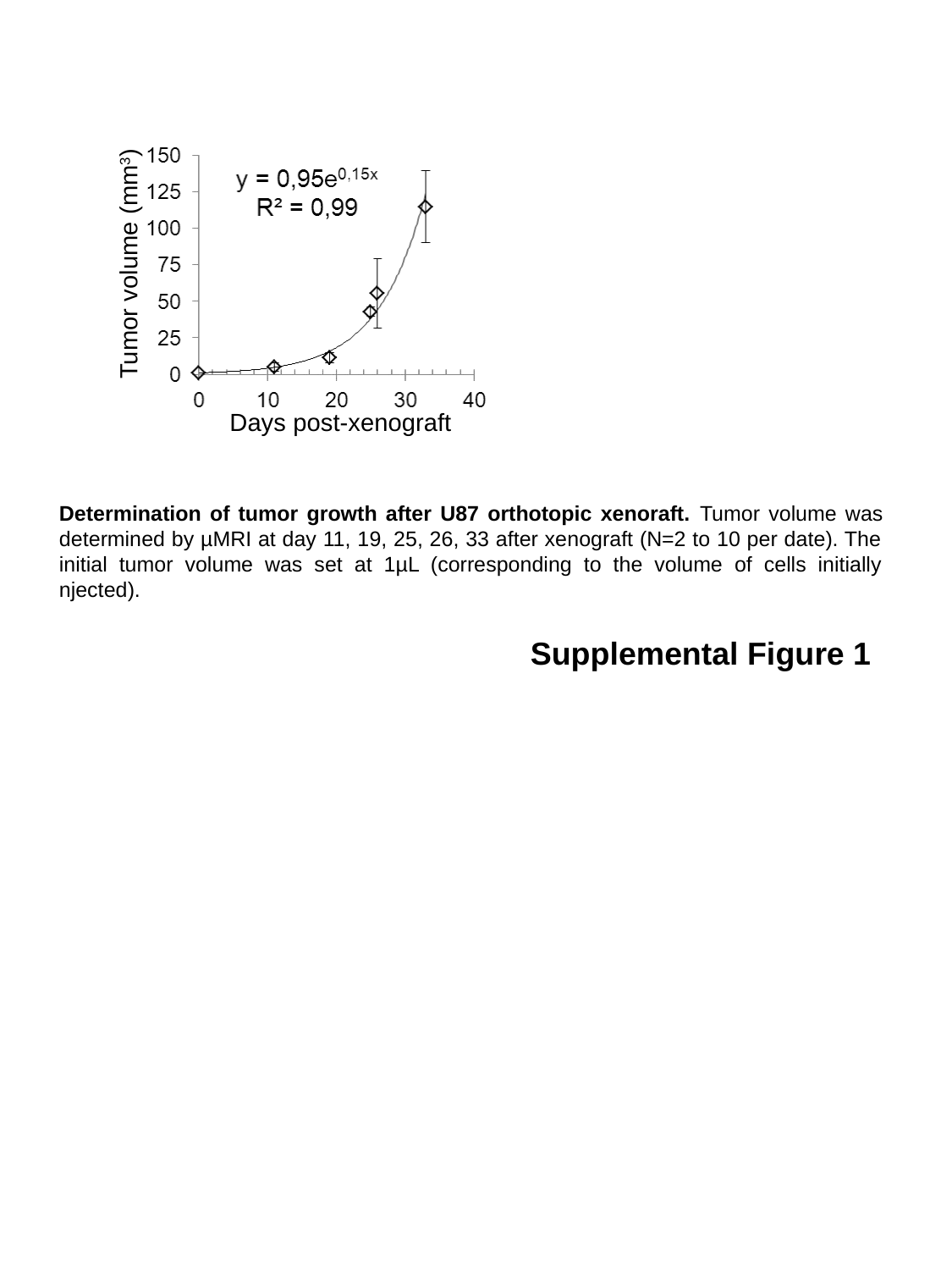

Tumor volume (mm3)
Days post-xenograft
Determination of tumor growth after U87 orthotopic xenoraft. Tumor volume was determined by µMRI at day 11, 19, 25, 26, 33 after xenograft (N=2 to 10 per date). The initial tumor volume was set at 1µL (corresponding to the volume of cells initially njected).
Supplemental Figure 1
